# iASPP induces EMT and cisplatin resistance in human cervical cancer through miR-20a-FBXL5/BTG3 signaling

**DOI:** 10.1186/s13046-017-0520-6

**Published:** 2017-04-11

**Authors:** Ying Xiong, Fei Sun, Peixin Dong, Hidemichi Watari, Junming Yue, Min-fei Yu, Chun-yan Lan, Yin Wang, Ze-biao Ma

**Affiliations:** 1 Department of Gynecology, State Key Laboratory of Oncology in South China, Sun Yat-sen University Cancer Center, Guangzhou, 510060 China; 2 Department of Obstetrics and Gynecology, Nanfang Hospital, Southern Medical University, Guangzhou, 510515 China; 3 Department of Women’s Health Educational System, Hokkaido University, Sapporo, 0608638 Japan; 4 Department of Gynecology, Hokkaido University School of Medicine, Hokkaido University, Sapporo, 0608638 Japan; 5 Department of Pathology and Laboratory Medicine, University of Tennessee Health Science Center, Memphis, TN 38163 USA; 6 Center for Cancer Research, University of Tennessee Health Science Center, Memphis, TN 38163 USA; 7 Guangzhou Sagene Biotech Co., Ltd, Guangzhou International Biotech Island, Guangzhou, 510300 China

**Keywords:** Cervical cancer, iASPP, EMT, Chemoresistance, FBXL5, BTG3

## Abstract

**Background:**

Epithelial–mesenchymal transition (EMT) and dysregulated microRNAs (miRNAs) have important roles in driving chemoresistance. We previously reported that iASPP is a key EMT inducer and could increase cisplatin resistance in cervical cancer (CC) cells. Herein, we investigate the downstream mechanisms through which iASPP contributes to EMT and cisplatin resistance in CC.

**Methods:**

By using a lentiviral system, we investigated the effects of iASPP knockdown on CC cell growth and chemosensitivity of CC cells to cisplatin in vivo. We examined if miR-20a, which was up-regulated following iASPP overexpression, would influence metastatic phenotypes and cisplatin resistance in CC cells, and explored the possible molecular mechanisms involved.

**Results:**

Knockdown of iASPP suppressed CC cell proliferation and sensitized CC cells to cisplatin in vivo. iASPP promotes miR-20a expression in a p53-dependent manner. Upregulation of miR-20a induced EMT and the recovery of CC cell invasion and cisplatin chemoresistance that was repressed by iASPP knockdown. We identified FBXL5 and BTG3 as two direct miR-20a targets. Silencing of FBXL5 and BTG3 restored cell invasion and cisplatin chemoresistance, which was suppressed by iASPP or miR-20a knockdown. Reduced FBXL5 and BTG3 expression was found in CC samples and associated with poor prognosis in CC patients.

**Conclusions:**

iASPP promotes EMT and confers cisplatin resistance in CC via miR-20a-FBXL5/BTG3 signaling.

**Electronic supplementary material:**

The online version of this article (doi:10.1186/s13046-017-0520-6) contains supplementary material, which is available to authorized users.

## Background

Cervical cancer (CC) ranks as the third most common female cancer worldwide, and is the leading cause of death from cancer among women in developing countries [1, 2]. Chemotherapy plays an important treatment option for CC [3, 4]. Cisplatin is a potent chemotherapeutic drug widely used for the treatment of numerous human cancers including CC, although its effectiveness is often limited by the development of resistance [5].

The protein iASPP, encoded by *PPP1R13L* gene, is overexpressed in human tumors [6], and can inhibit the function of p53 through inhibiting the transactivation function of p53 on the promoters of pro-apoptotic genes [7–9]. Inhibition of iASPP was shown to improve the efficacy of chemotherapy in cancer treatment [10–13]. Epithelial–mesenchymal transition (EMT) plays a key role in facilitating cancer metastasis, and suppression of EMT leads to enhanced sensitivity to chemotherapy [14]. We recently identified iASPP as a novel determinant of EMT and cisplatin resistance in CC cells [15]. However, the precise downstream mechanism through which iASPP contributes to EMT and cisplatin resistance in CC is unknown.

In this study, we showed that stable silencing of iASPP expression enhances cisplatin chemosensitivity in vivo, and miR-20a-FBXL5/BTG3 signaling is responsible for iASPP-induced EMT and cisplatin resistance. Our results uncovered a molecular basis for silencing iASPP to improve the activity of cisplatin, potentially providing a new therapeutic approach for human CC.

## Methods

### Human CC samples

After informed consent, 40 pairs of primary CC specimens and adjacent non-tumor cervical tissues were collected according to an Institutional Review Broad-approved protocol at the Sun Yat-Sen University Cancer Center (Guangzhou, China). Samples were snap-frozen and stored in liquid nitrogen until the RNA was extracted.

### Cell lines, culture condition and reagents

CC cell lines (HeLa and SiHa, ATCC), human embryonic kidney 293 T (HEK293T, ATCC) cells and immortalized human normal endometrial epithelial cell line (EM) [16] were maintained in DMEM/F12 medium (Gibco Laboratories, Grand Island, NY, USA) containing 10% fetal bovine serum (FBS; Gibco Laboratories, Grand Island, NY, USA). cisplatin (Sigma, St Louis, MO, USA) was used at a final concentration of 2.0 μg/ml. The miR-20a mimics (mimic-20a) or negative control mimic (mimic-NC), anti-miR-20a inhibitor (anti-20a), negative control inhibitor (anti-NC), siRNA targeting FBXL5 (AM16708) and BTG3 (AM16708) and respective negative controls (Thermo Fisher Scientific, Waltham, MA, USA) were transfected using Lipofectamine 2000 transfection reagent (Invitrogen, CA, USA). The iASPP expression vector, p53 expression vector and empty control vector (OriGene, MD, USA) were transfected using the Lipofectamine Plus reagent (Invitrogen, CA, USA).

### Plasmid construction and transfection

Lentiviral expression vector pLVX-shRNA and Lenti-X HTX packaging System were from Clontech (Palo Alto, CA, USA). Three different short hairpin RNA (shRNA) sequences (shRNA-iASPP-1, -2 and -3) targeting the coding region of *iASPP* gene and a negative control sequence, as listed in Table S1, were designed for by online design software BLOCK-iT™ RNAi Designer (Invitrogen Life Technologies, Carlsbad, CA, USA). Oligonucleotides were annealed and cloned into pLVX-shRNA vector sites (EcoRI and BamHI) to generate pLVX-shRNA-iASPP (shRNA-iASPP-1, -2 and -3) vectors and pLVX-shRNA-NC vector (shRNA-NC). The shRNA-iASPP vectors or shRNA-NC vector were co-transfected with the Lenti-X HTX Packaging Mix into HEK293T cells. HeLa and SiHa cells were infected with lentivirus supernatant at a multiplicity of infection (MOI) of 10, along with 5 μg/ml polybrene (Sigma-Aldrich, St Louis, MO, USA). After the fresh media change, infected cells were selected with 1 μg/mL of puromycin (Sigma-Aldrich, St Louis, MO, USA) for 3 weeks. After selection, western blotting was performed to determine if the knockdown was effective.

### Cell Counting Kit-8 (CCK8) Assay

Cells were seeded in 96-well plates (4000 cells/well). Twenty-four hours after seeding, cisplatin was added to cells. Cells were then incubated for 48 h with cisplatin, and cell viability was assessed using the Cell Counting Kit-8 assay according to the manufacturer’s protocol (Dojindo laboratories, Kumamoto, Japan). Relative survival was calculated as the ratio normalized to DMSO-treated controls (set as 1).

### RNA isolation and qPCR

Total RNA was extracted using TRIzol (Invitrogen, CA) according to the manufacturer’s protocol. For mRNA and mature miRNA analysis, cDNA was synthesized using the Thermo Scientific RevertAid First Strand cDNA Synthesis Kit (Thermo Fisher Scientific, Waltham, MA, USA) according to the manufacturer’s instructions. The mRNA levels were determined using Maxima™ SYBR Green/ROX qPCR Master Mix (2X) (Thermo Fisher Scientific, Waltham, MA, USA) on ABI 7500 Real-Time PCR Systems. Primers specific to FBXL5, BBTG3, E-cadherin and Vimentin were from the PrimerBank Web-based database (http://pga.mgh.harvard.edu/primerbank/). Detection of mature miRNA was performed using the NCode miRNA qRT–PCR kit (Invitrogen, CA), forward primers (the exact sequences of the mature miRNA genes) and a universal reverse primer supplied by the manufacturer. GAPDH and U6 small nuclear RNA were used as internal normalization controls. Data are expressed as the fold change over control (set as 1).

### Western blotting

Total cell lysates were extracted using the M-Per Mammalian Protein Extraction Reagent (Pierce Biotechnology, MA, USA), followed by immunoblotting with anti-iASPP (1:1000; Santa Cruz Biotechnology, Santa Cruz, CA, USA), p53 (1:1000; Santa Cruz Biotechnology, Santa Cruz, CA, USA), anti-phosphorylated p53 (1:1000; Abcam, Cambridge, UK), anti-Caspase-3 (Cell Signaling Technology, Danvers, MA, USA), anti-E-cadherin (1:1000; Cell Signaling Technology, Danvers, MA, USA), anti-Vimentin (1:1000, Cell Signaling Technology, Danvers, MA, USA), anti-FBXL5 (1:1000; Abcam, Cambridge, MA, USA), anti-BTG3 (1:1000, Abcam, Abcam, Cambridge, UK) or anti-GAPDH (1:2000; Santa Cruz Biotechnology, Santa Cruz, CA). Membranes were incubated with appropriate secondary antibodies and immunoreactive bands were visualized with ECL reagent (Perkin Elmer, Waltham, MA, USA). GAPDH was used as the endogenous control.

### In vitro invasion assay

Cell invasion assays were performed using a 24-well transwell inserts (Corning Costar, Cambridge, MA, USA) as previously described [17]. Briefly, 5 × 10^4^ cells in serum-free media were seeded in the upper chamber of a 24-well plate. The membrane of the Transwell unit was coated with Matrigel, and medium containing 10% FBS was added to in the lower compartment of each well. After 24 h, the non-motile cells on the upper surface of the inset were removed by a cotton swab. Invaded cells on the lower surface of the insert membrane was fixed and stained by Giemsa. The cells from at least five representative fields were counted. Relative invasion was expressed as the fold-change over the respective control.

### ChIP-qPCR assays

ChIP-qPCR analysis was performed using a Pierce Agarose ChIP kit (Pierce, Thermo Scientific, Rockford, IL, USA) according to the manufacturer’s instructions. Briefly, cells were cross-linked for 10 min with 1% formaldehyde and lysed. Protein-DNA complexes were immunoprecipitated using either anti-p53 antibody (Santa Cruz Biotechnology, Santa Cruz, CA, USA) or unrelated rabbit IgG (Thermo Fisher Scientific, Rockford, IL) as a negative control. The immunoprecipitated DNA was reverse cross-linked, purified and analyzed by quantitative real-time PCR, using primers specific for the *p21* [18], *miR-20a* [19] and *GAPDH* [20] genomic loci. Results are shown as the fold enrichment compared with IgG control, and further normalized to the *GAPDH* promoter.

### Microarray analysis

HeLa cells were transfected with anti-20a or anti-NC for 72 h. Total RNA was labeled and subsequently hybridized to the Superprint G3 Human GE 8x60k Microarray (Agilent Technologies) as described before [21]. Microarray expression data was imported into the GeneSpring software (Agilent Technologies). The differentially expressed mRNAs (*P* <0.05 and fold change ≥ 2.0) were selected for further analysis.

### Dual 3’-UTR reporter assay

Human FBXL5 or BTG3 3’-untranslated region (3’-UTR) cloned downstream of the firefly luciferase gene was purchased from OriGene Technologies (Rockville, MD, USA). Mutations in the miR-20a seed-matching sequences were created using a quick-change site-directed mutagenesis kit (Stratagene, CA, USA). Cells were seeded in 24-well plates and transfected after 24 h with 100 ng of the indicated firefly reporter vectors, 10 ng of Renilla reporter plasmid pRL-CMV as a normalization control, and 30 nM of mimic-20a or anti-20a or their negative controls. After 48 h, a Dual Luciferase Reporter assay (Promega, Madison, WI, USA) was performed according to the manufacturer’s instructions.

### Xenograft experiments

Female BALB/c-nu mice (aged 4 weeks) were purchased from Guangdong Medical Laboratory Animal Center (Guangzhou, China). All animal procedures were performed in accordance with Sun Yat-Sen University Cancer Center Institutional Animal Care and Use Committee guidelines. In brief, iASPP shRNA- or control shRNA-expressing HeLa cells (5 × 10^6^) were subcutaneously injected into the right flank of nude mice under sterile conditions. After the formation of palpable tumors (tumor volume reached 100 mm^3^), the mice were randomly divided into two groups with four animals in each group: PBS or cisplatin (5.0 mg/kg), via intraperitoneal injection twice a week for 24 days. Tumor sizes were measured every 6 days, and tumor volume was calculated by the formula: tumor volume (mm^3^) = width (mm^2^) × length (mm) × 0.5. After 24 days, all mice were sacrificed and photographed. Tumors were harvested and weighed.

### Statistics

Data are presented as mean ± SD. Differences were analyzed by a 2-tailed Student’s *t*-test or 1-way ANOVA. Calculations were performed using SPSS software (Release 13.0, Chicago, USA), and *P*-values of <0.05 were considered as significant.

## Results

### Stable knockdown of iASPP suppresses cisplatin resistance in vivo

**Fig. 1:**
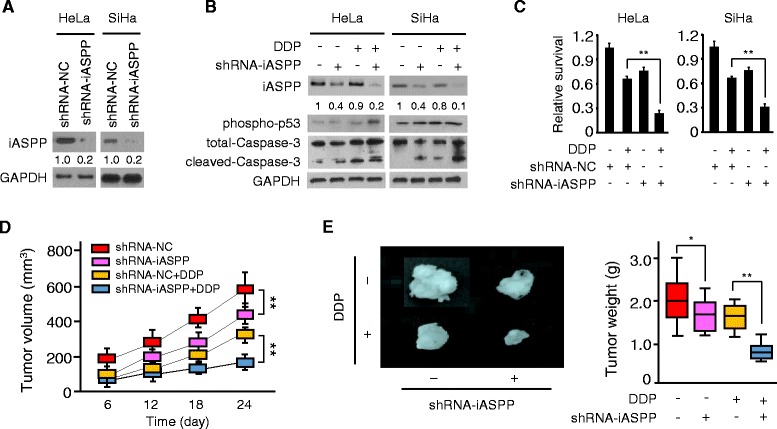
Stable knockdown of iASPP suppresses cisplatin resistance in vivo. **a** Western blot analysis of the iASPP protein in control (NC) shRNA or iASPP shRNA stably expressing CC cells. Quantitative analysis of iASPP protein expression (as expressed as ratio to GAPDH) is shown below blots. **b** Immunoblot of the indicated proteins in control or iASPP-silenced CC cells upon exposure to cisplatin (DDP). Quantitative analysis of iASPP protein expression (as expressed as ratio to GAPDH) is shown below blots. **c** Relative cell survival of control or iASPP-silenced CC cells treated with (or without) cisplatin, as assessed by CCK8 assays. **d** Volume of subcutaneous control or iASPP-silenced HeLa xenograft during 24 days of therapy with vehicle or cisplatin. **e** Representative tumors isolated from nude mice (*left*) and tumor weights (*right*) of HeLa subcutaneous tumor model treated with (or without) cisplatin. *P < 0.05; **P < 0.01

Recently we reported that elevated iASPP expression contributes to cisplatin resistance and EMT properties in CC cells [15]. To validate the role of iASPP as a promoter of chemoresistance and CC progression in vivo, we stably silenced endogenous iASPP in HeLa and SiHa cells with the transduction of shRNAs targeting iASPP. We used western blotting analysis to confirm the silencing effects of these shRNAs in CC cells. Transfection with all these shRNA-iASPP vectors led to significantly lower iASPP protein levels than transfection with shRNA-NC vector (Fig. 1a; Additional file 1: Figure S1A). We identified shRNA-iASPP-1 vector that produced the highest knockdown efficiency and therefore used it for further studies.

As shown in Fig. 1b, HeLa or SiHa cells with stable iASPP silencing exhibited reduced iASPP protein expression and enhanced protein expression of phospho-p53 and cleaved-Caspase-3, compared with control cells that were infected with control shRNA, indicating that stable silencing of iASPP reactivates p53 activity and promotes apoptosis in CC cells.

We next treated HeLa and SiHa cells with cisplatin, and found that upon cisplatin treatment, iASPP shRNA-transduced CC cells had increased levels of phospho-p53 and cleaved-Caspase-3 compared to control shRNA-transduced cells, as demonstrated by Western blotting (Fig. 1b). CCK8 assay confirmed that iASPP shRNA-expressing CC cells had significantly decreased cell survival (up to 50%) in response to cisplatin, compared with control shRNA-transduced cells (Fig. 1c), suggesting that stable depletion of iASPP sensitized the drug response of CC cells to cisplatin.

Then we examined whether iASPP silencing could enhance the sensitivity of CC cells to cisplatin in vivo, using a human CC xenograft model generated from HeLa cells. In the absence of cisplatin treatment, there was a moderate (nearly 30%) decrease in tumor volume and weight of iASPP knockdown group compared with control group (Fig. 1d and e). However, when cisplatin was administered at a dose of 5.0 mg/kg, mice injected with iASPP shRNA-expressing HeLa cells showed a more dramatic (approximately 50%) reduction in tumor volume and tumor weight, compared with mice injected with control shRNA-expressing cells (Fig. 1d and e). Thus, stable iASPP knockdown reverses the resistance of the human CC xenograft model to cisplatin treatment in vivo.

### iASPP induces miR-20a expression in a p53-dependent manner

**Fig. 2:**
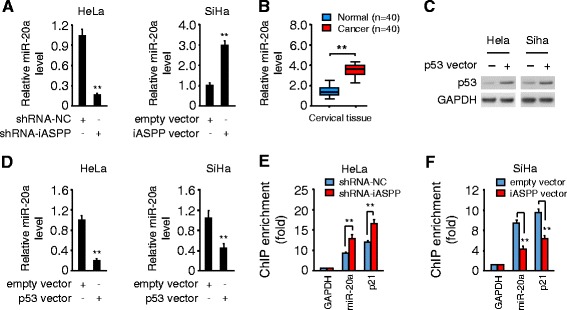
iASPP induces miR-20a expression in a p53-dependent manner. **a** qPCR analysis of miR-20a in control or iASPP-silenced HeLa cells and in SiHa cells after transient overexpression of iASPP. **b** Relative miR-20a expression in 40 paired cancerous and normal tissue samples from CC patients, measured by qPCR analysis. **c** Protein expression of p53 in CC cells transfected with control or p53 expression vector (p53-vec). **d** Relative miR-20a expression in CC cells after transient overexpression of p53. **e**, **f** ChIP-qPCR assay of p53 occupancy at the human miR-20a and *p21* (as a control) loci in control or iASPP-silenced HeLa cells (**e**), and in SiHa cells following transient overexpression of iASPP (**f**). **P < 0.01

Since p53 has also been shown to directly repress the oncogenic miR-17-92a cluster (comprising miR-17, miR-18a, miR-20a, miR-19a, miR-19b and miR-92a) through binding the TATA box of the miR-17-92 promoters [19], we hypothesized that, via a p53-depedent mechanism, iASPP may promote cisplatin resistance by inducting this cluster, which was shown to enhance chemoresistance in mantle cell lymphoma [22]. We examined the levels of these six miRNAs in iASPP shRNA or control shRNA-expressing HeLa cells. Indeed, stable iASPP silencing in HeLa cells resulted in a pronounced repression of miR-20a (Fig. 2a) and a moderate repression of the remaining five miRNAs (data not shown). Transient transfection of iASPP expression vector in SiHa cells significantly increased miR-20a expression (Fig. 2a). As shown in Fig. 2b, miR-20a was markedly upregulated in human CC samples compared with the paired normal cervical tissues, as determined by qPCR. Moreover, we observed the inhibitory effects of ectopic p53 expression on miR-20a level by qPCR in CC cells (Fig. 2c and d). These data indicate that iASPP may induce the expression of miR-20a via modulating p53 activity in CC cells.

Further ChIP-qPCR assays revealed that p53 selectively bond to the promoters of miR-20a and p21 (positive control for the assay) (Fig. 2e and f). Consistent with a role of iASPP in suppressing the transcriptional functions of p53 [15], knockdown of iASPP in HeLa cells significantly increased the p53 occupancy at the miR-20a promoter, while enforced expression of iASPP in SiHa cells triggered a release of p53 from the miR-20a promoter (Fig. 2e and f). These results indicate that iASPP prevents p53 recruitment to the miR-20a promoter, leading to the induction of miR-20a in CC cells.

### miR-20a mediates iASPP-induced EMT and cisplatin resistance

**Fig. 3:**
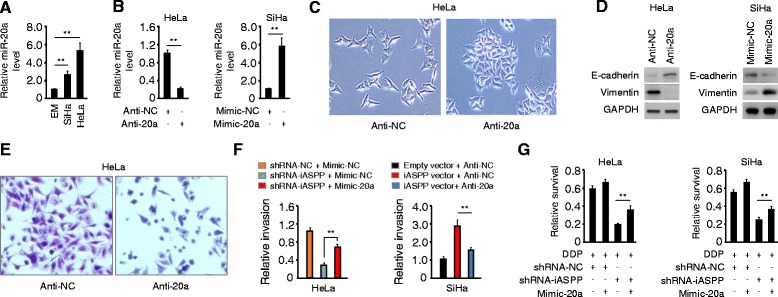
miR-20a mediates iASPP-induced EMT and cisplatin resistance. **a** Relative miR-20a expression in CC cells and normal endometrial epithelial cell EM, as measured by qPCR analysis. **b** qPCR analysis of miR-20a in HeLa cells transfected with anti-miR-20a inhibitor (anti-20a) or negative control inhibitor (anti-NC), or in SiHa cells transfected with miR-20a mimic (mimic-20a) or negative control (mimic-NC). **c** Morphology of HeLa cells transfected with anti-20a or anti-NC. **d** Immunoblot of the indicated proteins in CC cells after knockdown or overexpression of miR-20a. **e** Representative image of invaded Hela cells in cell invasion assay. **f** Relative invasion of indicated CC cells after overexpression or knockdown of miR-20a. **g** CC cells transfected with mimic-20a or mimic-NC were treated with cisplatin, and relative cell survival was analyzed by CCK8 assays. **P < 0.01

qPCR analysis of CC cell lines and immortalized normal epithelial cell line EM demonstrated the up-regulation of miR-20a in CC cells (Fig. 3a). We next sought to determine the role of miR-20a in regulating iASPP-induced EMT and cisplatin resistance by modulating its levels in CC cells. Transient transfection of HeLa cells with anti-20a promotes an epithelial cellular phenotype, as evidenced by a transition from the mesenchymal phenotype to an epithelial cobblestone phenotype (Fig. 3b and c). Given the morphological changes observed in HeLa cells, we evaluated whether these changes are associated with changes in EMT markers. Western blotting data showed that HeLa cells transfected with anti-20a displayed an upregulation of E-cadherin and a repression of Vimentin, compared with control cells transfected with anti-NC (Fig. 3d). Knockdown of miR-20a also resulted in decreased invasiveness of HeLa cells (Fig. 3e). In contrast, SiHa cells transfected with mimic-20a displayed a downregulation of E-cadherin and an induction of Vimentin, compared with control cells transfected with mimic-NC (Fig. 3b and d).

Of note, enforced miR-20a expression partially rescued the effects of iASPP knockdown by sustaining invasiveness of HeLa cells (Fig. 3f), whereas anti-20a-mediated downregulation of miR-20a significantly attenuated iASPP-driven invasion of SiHa cells (Fig. 3f). CCK8 assay demonstrated that overexpression of miR-20a significantly decreased the activity of cisplatin by restoring cell survival, which was suppressed by iASPP depletion (Fig. 3g). These data suggest that miR-20a is required for iASPP-induced invasion and cisplatin resistance in CC cells.

### miR-20a-FBXL5/BTG3 signaling is responsible for iASPP-induced cell invasion and cisplatin resistance

**Fig. 4:**
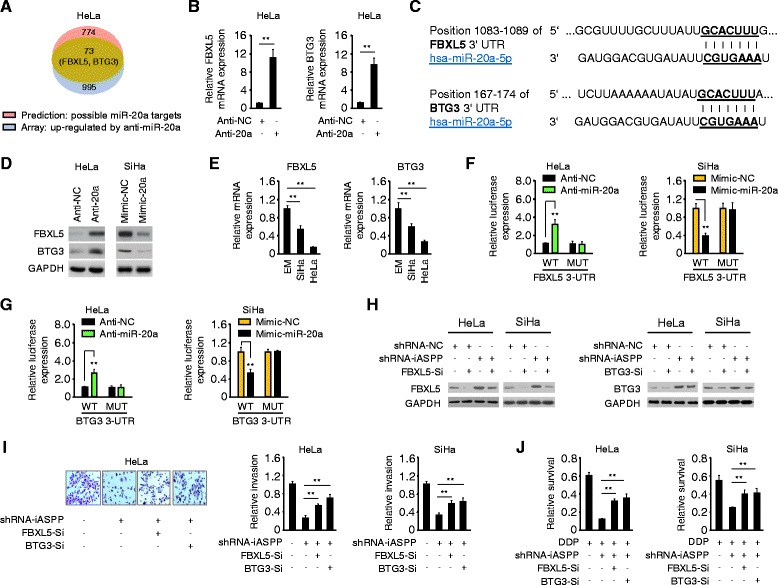
miR-20a-FBXL5/BTG3 signaling is responsible for iASPP-induced cell invasion and cisplatin resistance. **a** Venn diagram depicting 73 genes (including FBXL5 and BTG3) that were predicted potential targets of miR-20a, and were up-regulated by anti-20a in HeLa cells. **b** qPCR analysis of FBXL5 and BTG3 expression in CC cells after knockdown or overexpression of miR-20a. **c** Schematic representation of the 3’-UTR of FBXL5 or BTG3 with the predicted target site for miR-20a. **d** Expression of FBXL5 or BTG3 protein in CC cells after the transient knockdown or overexpression of miR-20a. **e** qPCR analysis of FBXL5 or BTG3 expression in CC cell lines and a normal cell line EM. **f**, **g** HeLa and SiHa cells were co-transfected with reporter plasmids containing the wild-type FBXL5 (**f**)/BTG3 (**g**) or a mutant FBXL5/BTG3 3’-UTR, together with a miR-20a mimic, miR-20a inhibitor or respective negative controls. The relative luciferase activity was assayed. **h** Immunoblot of FBXL5 (*left*) or BTG3 (*right*) protein expression in control or iASPP-silenced CC cells transfected with FBXL5 siRNA (FBXL5-Si), BTG3 siRNA (BTG3-Si) or control siRNA. **i** Representative images of invaded Hela cells (*left*) and relative invasion of control or iASPP-silenced CC cells after knockdown of FBXL5 or BTG3 (*right*). **j** Relative cell survival of control or iASPP-silenced CC cells after knockdown of FBXL5 or BTG3, upon exposure to cisplatin, as assessed by CCK8 assays. **P < 0.01

To find potential target genes affected by miR-20a, we performed a microarray gene expression analysis in HeLa cells transfected with anti-20a. This analysis revealed that more than 1000 genes were up-regulated by miR-20a knockdown. Using this list, we performed online miRDB target gene prediction analysis [23], and narrowed the predicted targets of miR-20a down to 73 common genes (Fig. 4a; Additional file 1: Table S2). Among these, FBXL5 and BTG3 have been reported to regulate EMT and cancer metastasis [24–26]. Data from the microarray analysis was confirmed by qPCR in HeLa cells (Fig. 4b). We identified putative binding sites for miR-20a in the 3’-UTRs of FBXL5 and BTG3 (Fig. 4c). As expected, transfection with anti-20a increased FBXL5 and BTG3 protein levels in HeLa cells, however miR-20a re-expression decreased FBXL5 and BTG3 protein levels in SiHa cells (Fig. 4d). Consistent with these data, a negative correlation between endogenous miR-20a levels and FBXL5 and BTG3 mRNA expression was found in CC cells, as measured by qPCR (Figs. 3a and 4e). Luciferase assay showed that anti-20a enhanced the activity of the FBXL5 or BTG3 3’-UTR, and mimic-20a significantly decreased the activity of the FBXL5 or BTG3 3’-UTR (Fig. 4f and g). Importantly, miR-20a expression did not alter the activity of luciferase linked to FBXL5 or BTG3 3’-UTR where the putative miR-20a binding site was mutated (Fig. 4f and g). Thus, the FBXL5 and BTG3 mRNA are directly regulated by miR-20a in CC cells.

We next asked whether miR-20a-FBXL5/BTG3 signaling is implicated in iASPP-induced cell invasion and cisplatin resistance in CC cells. iASPP- or miR-20a-silenced CC cells had increased protein levels of FBXL5 and BTG3, compared with respective control cells (Fig. 4h; Additional file 1: Figure S1B). We found that siRNA-mediated knockdown of FBXL5 or BTG3 significantly restored the invasive abilities and reduced the anti-tumor activity of cisplatin in iASPP- or miR-20a-silenced CC cells (Fig. 4i and j; Additional file 1: Figure S1C). Furthermore, the knockdown of FBXL5 or BTG3 resulted in E-cadherin inhibition and recovery of Vimentin in iASPP- or miR-20a-silenced cells (Additional file 1: Figure S1D and not shown). Taken together, these data indicate that FBXL5 and BTG3 serve as tumor suppressors in CC cells, and miR-20a-FBXL5/BTG3 signaling is responsible for iASPP-induced EMT and cisplatin resistance.

### High iASPP and low FBXL5/BTG3 expression correlates with poor survival in CC patients

**Fig. 5:**
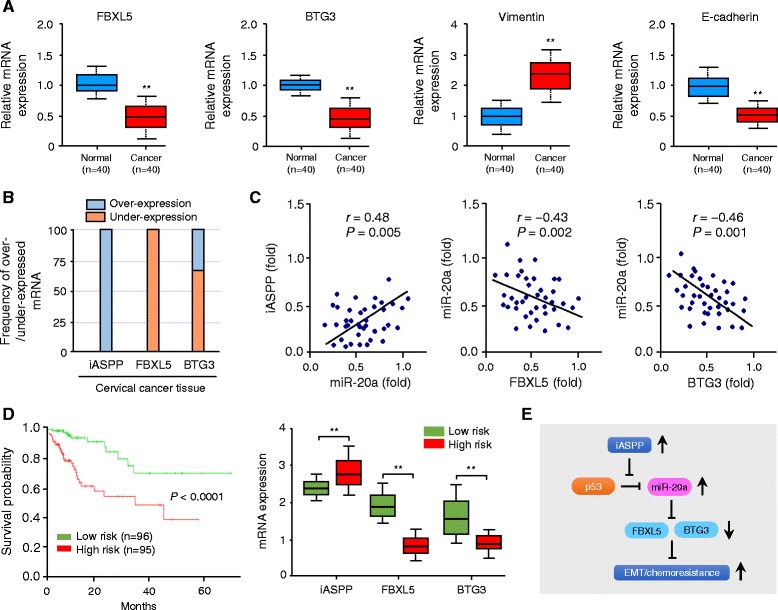
High iASPP and low FBXL5/BTG3 expression correlates with poor survival in CC patients. **a** The mRNA expression of FBXL5, BTG3, Vimentin and E-cadherin was assessed by qPCR analysis in 40 paired cancerous and normal tissue samples of CC patients. **b** Pan-cancer analysis of iASPP, FBXL5 and BTG3 mRNA expression in human cervical cancer tissues and adjacent normal tissues was performed using the BioExpress database. **c** Correlative analysis of the indicated mRNAs and miR-20a in 40 paired tumor and non-tumor tissues of CC patients. The expression of mRNAs and miR-20a was determined using qPCR. Relative expression of each mRNA and miR-20a in tumor tissues was calculated as a fold change compared to that of corresponding non-tumor tissues. Spearman correlation coefficient with the respective significance is shown. **d** Kaplan-Meier survival curve of 191 TCGA (Cancer Genome Atlas database) cervical cancer samples was created using the SurvMicro database, based on the low or high risk for a poor outcome (*left*). Box plots demonstrating significantly higher level of iASPP and lower level of FBXL5/BTG3 in the high-risk CC patients (*right*). **e** Schematic representation of the proposed mechanism by which iASPP promotes EMT and cisplatin resistance in CC via miR-20a-FBXL5/BTG3 signaling. **P < 0.01

Given that iASPP and miR-20a expression is elevated in CC tissues [15] (Fig. 2b), we assessed whether FBXL5 and BTG3 are differentially expressed in primary CC samples using qPCR analysis. As shown in Fig. 5a, lower expression of FBXL5, BTG3 and E-cadherin along with higher Vimentin level was detected in CC samples compared with the paired normal cervical tissues. Then we performed a pan-cancer bioinformatics analysis based on the BioXpress database [27], to analyze the mRNA expression of FBXL5 and BTG3 in cancer tissues and their paired normal tissues across multiple cancer types. Interestingly, iASPP is up-regulated in 100% of CC tissues, however FBXL5 and BTG3 are down-regulated in 100 and 67% in CC tissues, respectively (Fig. 5b). This analysis also indicated a similar negative correlation between high iASPP and low FBXL5/BTG3 expression in various cancer types (Additional file 1: Figure S2).

To test whether the iASPP-miR-20a-FBXL5/BTG3 signaling described above is clinically relevant, we performed qPCRs to assess correlations between the abundance of iASPP, miR-20a, FBXL5 and BTG3 in 40 paired tumor and non-tumor tissues. We could detect a significant positive correlation between iASPP levels and the expression of miR-20a, and a significant negative association of miR-20a and FBXL5/BTG3 mRNA expression (Fig. 5c). These results show that the iASPP-miR-20a-FBXL5/BTG3 signaling is active in primary human CCs.

Finally, we examined the association between FBXL5/BTG3 and patient survival using the SurvExpress web tool [28]. SurvExpress uses the Cox model and TCGA RNA sequencing data to give each sample a risk score, and CC patients were divided into the high-risk (with a low probability of survival; above median of risk score; *n* = 95) or low-risk (with a high probability of survival; below the median of risk score; *n* = 96) group (Fig. 5d). Kaplan–Meier survival analysis demonstrated that the overall survival rate in the high-risk group were significantly lower than those in the low-risk group (*P* < 0.001) (Fig. 5d). High-risk CC patients exhibited higher iASPP and lower FBXL5/BTG3 expression than the low-risk patients (Fig. 5d).

These data, together with those outlined above, support a model that: through a p53-depedent mechanism, iASPP up-regulates miR-20a expression, leading to the repression of FBXL5 and BTG3 expression, which in turn promotes EMT, cell invasion and cisplatin resistance in CC (Fig. 5e).

## Discussion

Resistance to chemotherapy is a major obstacle for effective treatment of cancers including CC [29]. The acquisition of EMT features has been proposed as the key contributor of chemoresistance in cancer cells [30, 31]. In CC, EMT was reported to play a major role in promoting chemoresistance [32]. Hence, it is crucial to obtain better insight into the mechanisms underlying the induction of EMT and to explore novel approaches to improve drug sensitivity in CC patients. Although iASPP was known to regulate EMT in CC cells [15] and overexpression of iASPP correlates with poor prognosis and chemoresistance in CC [33], the underlying mechanism are not understood.

In this study, we demonstrate that stable silencing of iASPP sensitizes CC cells to cisplatin-induced cell death in vivo, supporting iASPP as a new therapeutic candidate to overcome cisplatin resistance in CC. Furthermore, we provided the first evidence that iASPP stimulates EMT and induces cisplatin resistance in both cervical adenocarcinoma cell line HeLa and cervical squamous cell carcinoma cell line SiHa, through regulating the downstream miR-20a-FBXL5/BTG3 signaling pathway.

The miR-17-92 cluster is highly expressed in various cancer types, including B-cell lymphoma [34], breast cancer [35], colon cancer [36], lung cancer [37] and CC [38, 39]. Among this cluster, miR-20a is a known oncogenic miRNA, thought be critically associated with enhanced EMT properties in colon cancer [40] and cisplatin-resistant phenotype in gastric cancer [41]. In CC cells, overexpression of miR-20a facilitates the proliferation and metastasis of CC [39]. Our results validated invasion-promoting role of miR-20a in CC cells, and further emphasized the requirement of miR-20a in iASPP-induced EMT and cisplatin resistance. Therefore, inactivation of miR-20a might be a novel strategy for the treatment of CC.

Overexpression of miR-17-92 cluster can be attributable to the amplification of the miR-17-92 locus [34], direct transactivation by c-Myc [42] or indirect Notch-dependent activation [43]. However, the molecular mechanism of miR-20a overexpression in CC remains unclear. Previous study showed that tumor suppressor p53 can directly bind to the promoters of miR-17-92 and repress its transcription [19]. In line with this finding, we found that, though interfering with the activity of p53, iASPP up-regulates miR-20a level in CC cells, leading to EMT features, increased cell invasion and resistance to cisplatin treatment.

FBXL5 inhibits EMT and attenuates the metastasis and cisplatin resistance in gastric cancer [24, 25]. BTG3 was also shown to suppress proliferation and invasion of gastric and esophageal cancer cells [26, 44]. We demonstrated for the first time that FBXL5 and BTG3 are down-regulated in CC tissues and their downregulation are associated with worse prognosis in CC patients. In CC cells, FBXL5 and BTG3 function as tumor suppressors and direct downstream targets of miR-20a, and the repression of FBXL5 and BTG3 can restore the invasive and resistant characteristics of CC cells, which was suppressed by iASPP or miR-20a depletion.

## Conclusions

In conclusion, our data suggest that iASPP promotes EMT and confers cisplatin resistance in CC via miR-20a-FBXL5/BTG3 signaling, and blocking iASPP activity or down-regulating its expression in CC may be a novel therapeutic strategy to improve the efficacy of cisplatin, one of the most widely used chemotherapy agents.

## Additional file

Additional file 1: Silencing of FBXL5 and BTG3 restored the effects that was suppressed by iASPP or miR-20a knockdown. (DOCX 1058 kb)

## Abbreviations

CC: Cervical cancer
DDP: Cisplatin
EMT: Epithelial-to-mesenchymal transition
miRNA: microRNA
