# Supplementary material for: iASPP induces EMT and cisplatin resistance in human cervical cancer through miR-20a-FBXL5/BTG3 signaling
Source: J Exp Clin Cancer Res. 2017 Apr 11;36:48. doi: 10.1186/s13046-017-0520-6 (PMC5387376; doi:10.1186/s13046-017-0520-6)
Supplement: Additional file 1: — Silencing of FBXL5 and BTG3 restored the effects that was suppressed by iASPP or miR-20a knockdown. (DOCX 1058 kb) [file 13046_2017_520_MOESM1_ESM.docx]

**Supplemental Information**

**iASPP induces EMT and cisplatin resistance in human cervical cancer through miR-20a-FBXL5/BTG3 signaling**

Ying Xiong, Fei Sun, Peixin Dong, Yosuke Konno, Sharon JB Hanley, Min-fei Yu, Chun-yan Lan, Yin Wang, Ze-biao Ma, Junming Yue and Hidemichi Watari

**
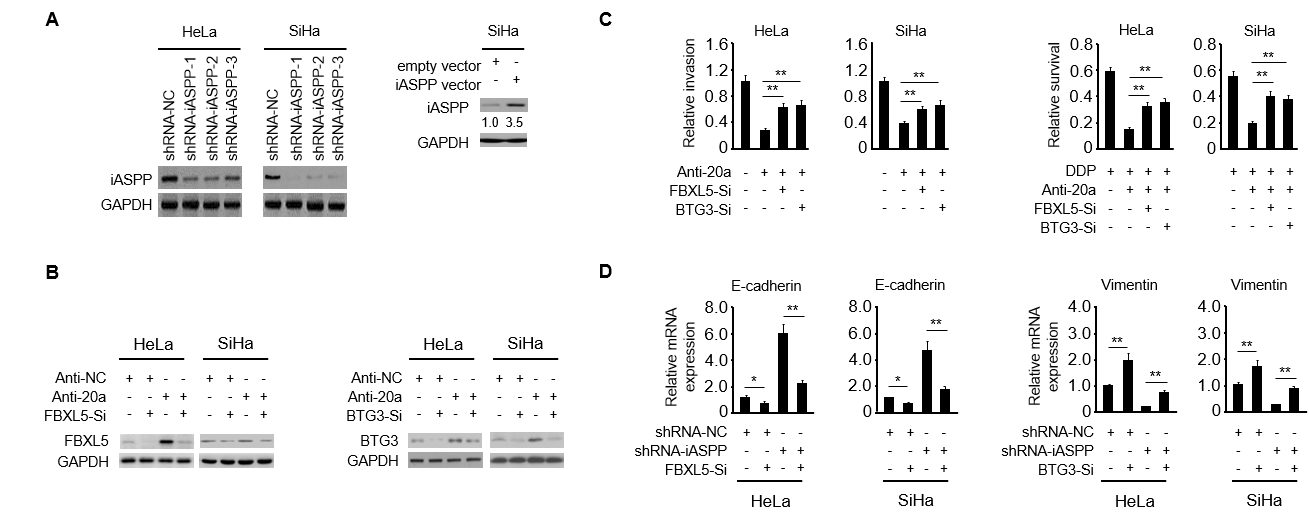
**

**Supplementary Figure 1: Silencing of FBXL5 and BTG3 restored the effects that was suppressed by iASPP or miR-20a knockdown.**

(**A**) Immunoblot of iASPP protein in control (NC) shRNA or iASPP shRNAs stably expressing CC cells (left panel). Quantitative analysis of iASPP protein expression (as expressed as ratio to GAPDH) in SiHa cell transfected with control or iASPP expression vector is shown below blots (right panel). (**B**) Immunoblot of FBXL5 (left) or BTG3 (right) protein in control or miR-20a-silenced CC cells after transient knockdown of FBXL5 or BTG3. (**C**) Relative invasion of control or miR-20a-silenced CC cells after knockdown of FBXL5 or BTG3 (left), and relative cell survival of control or miR-20a-silenced CC cells after knockdown of FBXL5 or BTG3 (right), upon exposure to DDP, as assessed by CCK8 assays. (**D**) qPCR analysis of E-cadherin (left) and Vimentin (right) in control or iASPP-silenced CC cells after transient knockdown of FBXL5 or BTG3. **P* < 0.05; ***P* < 0.01.

**
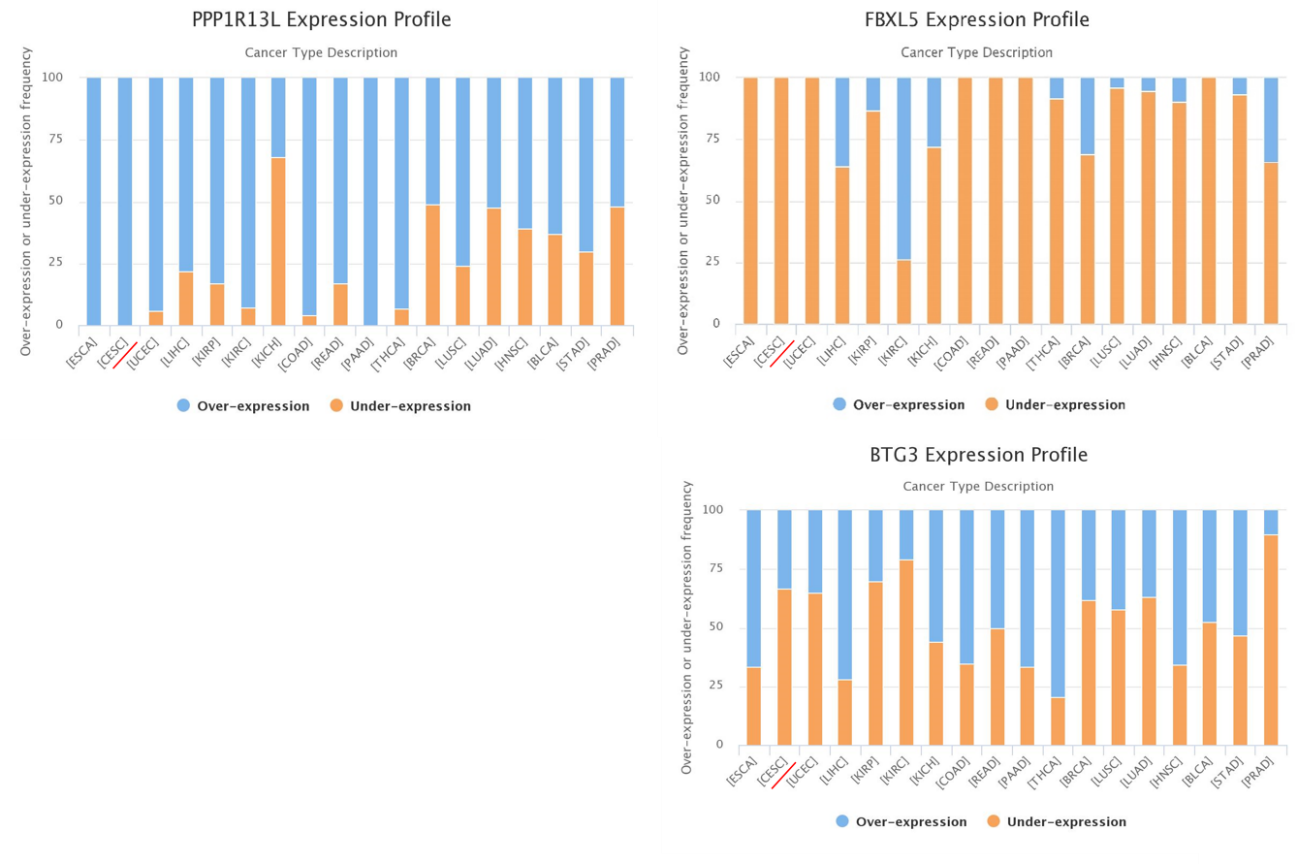
**

**Supplementary Figure 2: A negative correlation between high iASPP and low FBXL5/BTG3 expression in various cancer types**.

Pan-cancer analysis of iASPP, FBXL5 and BTG3 mRNA expression in paired human normal and cancerous tissues was performed using the BioExpress database. Note: iASPP (PPP1R13L); cervical squamous cell carcinoma (CESC).

**Supplementary Table 1. Targeting sequences of shRNA-iASPP and shRNA-NC.**

| **Name** | **target sequence** | **shRNA primers** |
| --- | --- | --- |
| shRNA-iASPP-1 | CCTCAAAGGAGTAAAGTCT | **F:** 5'-GATCCG**CCTCAAAGGAGTAAAGTCT**TTCAAGAGAAGACTTTACTCCTTTGAGGTTTTTTG-3'  **R:** 3'-GCGGAGTTTCCTCATTTCAGAAAGTTCTCTTCTGAAATGAGGAAACTCCAAAAAACTTAA-5' |
| shRNA-iASPP-2 | CGGCCAAGGTGGATGAACT | **F:** 5'-GATCCG**CGGCCAAGGTGGATGAACT**TTCAAGAGAAGTTCATCCACCTTGGCCGTTTTTTG-3'  **R:** 3'-GCGCCGGTTCCACCTACTTGAAAGTTCTCTTCAAGTAGGTGGAACCGGCAAAAAACTTAA-5 |
| shRNA-iASPP-3 | CAGAGCAGCCGCAGAGCAT | **F:** 5'-GATCCG**CAGAGCAGCCGCAGAGCAT**TTCAAGAGAATGCTCTGCGGCTGCTCTGTTTTTTG-3'  **R:** 3'-GCGTCTCGTCGGCGTCTCGTAAAGTTCTCTTACGAGACGCCGACGAGACAAAAAACTTAA-5' |
| shRNA-NC | TCACCGAGATGAAGATATC | **F:** 5'-GATCCG**TCACCGAGATGAAGATATC**TTCAAGAGAGATATCTTCATCTCGGTGATTTTTTG-3'  **R:** 3'-GCAGTGGCTCTACTTCTATAGAAGTTCTCTCTATAGAAGTAGAGCCACTAAAAAACTTAA-5' |

**Supplementary Table 2. Top 10 up-regulated mRNAs by anti-miR-20a inhibitor in human cervical cancer HeLa cell, measured with microarray analysis.**

| No. | Gene |
| --- | --- |
| 1 | *KATNAL1* |
| 2 | *ARID4B* |
| 3 | *PKD2* |
| 4 | *SLC40A1* |
| 5 | ***BTG3*** |
| 6 | *ZNF800* |
| 7 | *PTPN4* |
| 8 | *ZNFX1* |
| 9 | ***FBXL5*** |
| 10 | *EPHA4* |
